# Applying Machine Learning Techniques to Implementation Science

**DOI:** 10.2196/50201

**Published:** 2024-04-22

**Authors:** Nathalie Huguet, Jinying Chen, Ravi B Parikh, Miguel Marino, Susan A Flocke, Sonja Likumahuwa-Ackman, Justin Bekelman, Jennifer E DeVoe

**Affiliations:** 1 Department of Family Medicine Oregon Health & Science University Portland, OR United States; 2 BRIDGE-C2 Implementation Science Center for Cancer Control Oregon Health & Science University Portland, OR United States; 3 Section of Preventive Medicine and Epidemiology Department of Medicine Boston University Chobanian & Avedisian School of Medicine Boston, MA United States; 4 Data Science Core Boston University Chobanian & Avedisian School of Medicine Boston, MA United States; 5 iDAPT Implementation Science Center for Cancer Control Wake Forest School of Medicine Winston-Salem, NC United States; 6 Perelman School of Medicine University of Pennsylvania Philadelphia, PA United States; 7 Penn Center for Cancer Care Innovation, Abramson Cancer Center Penn Medicine Philadelphia, PA United States

**Keywords:** implementation science, machine learning, implementation strategies, techniques, implementation, prediction, adaptation, acceptance, challenges, scientist

## Abstract

Machine learning (ML) approaches could expand the usefulness and application of implementation science methods in clinical medicine and public health settings. The aim of this viewpoint is to introduce a roadmap for applying ML techniques to address implementation science questions, such as predicting what will work best, for whom, under what circumstances, and with what predicted level of support, and what and when adaptation or deimplementation are needed. We describe how ML approaches could be used and discuss challenges that implementation scientists and methodologists will need to consider when using ML throughout the stages of implementation.

## Introduction

Implementation science is a research field developing and testing methods and strategies that can improve the uptake of evidence-based interventions (EBIs) and practices into routine use in targeted settings [1]. It has important applications in both clinical and public health settings, such as health care facilities, public health departments, schools, and workplaces [2-4]. For example, the RE-AIM (Reach, Effectiveness, Adoption, Implementation, and Maintenance) framework, which was proposed by implementation scientists to guide the planning and evaluation of programs, has been used for health care– and community-based programs promoting chronic disease prevention and management, healthy aging, mental health, and health behavior change [5]. In addition, implementation science methods have been applied in clinical settings (eg, clinic-initiated cancer screening, tobacco cessation, and mental health programs) to scale up effective interventions to improve population health [4].

Implementation strategies are the methods, actions, and activities that aim to enhance the adoption, implementation, and sustainability of EBIs in clinical and public health practice. Implementation strategies can target multiple levels (eg, communities, hospitals, health care clinics, public health departments, clinical and public health practitioners, and individual patients and community members) and may involve multiple components (eg, information technology tools, workflow changes, and policies mandating services) and activities (eg, training and incentives) [6,7]. Numerous factors, such as target populations and targeted behavior change, varied uptake of strategies across settings, the actors that deliver the implementation strategies, and the timing of the EBI implementation, can influence the implementation processes and outcomes [6-8]. Further, there is often a need to tailor or adapt implementation strategies and the associated activities to the local, dynamic context to increase implementation success. Given the multifactorial drivers and their complex relationships, implementation science could benefit from advanced data analytics frameworks and methods for artificial intelligence and machine learning (ML).

As a subfield of artificial intelligence, ML [9,10] develops automated methods and algorithms that learn from data. With this learning, it can then perform tasks such as prediction and pattern discovery. To date, ML applications in health care settings have been focused on supplementing clinical work, predicting health-related outcomes (eg, disease severity and prognosis) [11-14], and supporting clinical decisions (eg, tailoring medications and other treatments) [15-17]. Applications of ML in public health include population health surveillance and outbreak mitigation, evaluating the effectiveness of public health strategies and campaign and disaster and emergency alleviation [18-22]. Existing literature on the application of ML in the field of implementation science is sparse [23]. However, ML has great potential to be applied in areas such as tailoring strategies and support activities, supporting decision-making on the selection of actors or settings, and predicting and understanding the impact of implementation strategies on the adoption of EBIs across different settings and target populations. The aim of this viewpoint is to introduce a roadmap for applying ML techniques to address implementation science questions, describe a few limited real-world applications of ML related to implementation science, and discuss challenges that implementation scientists and methodologists may face along the way when using ML as a strategy to monitor EBI adoption or to inform the need for interventions.

## A Roadmap for Applying ML in Implementation Science

ML approaches can be applied across the continuum of EBI implementation. Here, we use the strategic implementation framework (SIF) [24] as a roadmap to illustrate the potential application of ML at different stages of implementation, as summarized in Table 1. The SIF depicts 3 stages of implementation (ie, setting the stage; active implementation; and monitor, support, and sustain) and the distinct types of strategies needed for practice change in each stage to ensure that improvements are supported and sustained.

**Table 1:** Roadmap for implementation scientists and methodologists to use ML^a^.

	Strategic implementation framework stages		
	Setting the stage	Active implementation	Monitor, support, and sustain
Implementation goals and activities	Understand the local context to select implementation strategies and prepare for implementation	Implement strategies and support activities to improve EBI^b^	Monitor the sustained adoption of strategy and EBI improvements
Implementation challenges	Limited data available or used The context is not static End users with differing priorities	The context is not static End users with differing priorities Need to adapt to setting and targeted population	The context is not static (new guidelines, policy, and care delivery)
Opportunities for ML application	Predict who will adopt an EBI Determine the level of support needed Identify the need for change	ML as a strategy	Monitor progress Inform need for deimplementation Inform need for support
Considerations when using ML across the 3 stages	Setting characteristics Time period Data completeness Multilevel strategy	N/A^c^	Risk prediction bias Recalibration of ML Adaptation of ML Deimplementation of ML

^a^ML: machine learning.

^b^EBI: evidence-based intervention.

^c^N/A: not applicable.

### Setting the Stage

Setting the stage refers to preimplementation activities such as assessing readiness to change, identifying barriers and facilitators to implementing EBIs, selecting or developing strategies to support implementation, and identifying and acquiring resources. Implementation scientists often find that an effective strategy in one setting may not work well in other settings and that some may need more or different types of support (eg, hours of training, intensity of coaching support, or remote vs in-person training). As such, one of the biggest implementation science challenges is to identify what works, for whom, under what circumstances, and with what level of support.

Typical approaches for selecting and tailoring implementation strategies to fit the local context (eg, process mapping, intervention mapping, and coincidence analysis) address this challenge at the organization or population levels [25]. Often, data to inform the selection of an implementation strategy are limited to surveys, qualitative interviews, and organization-level data. However, clinical or public health data (eg, electronic health records [EHRs], administrative data, claims data, patient or disease registries, immunization registries, and health surveys), data linkages (eg, EHR data linked across practice sites, water quality, and air quality), and data related to implementation processes (eg, responses of patients, community members, and practitioners to a specific implementation science strategy from prior studies) are increasingly available. Implementation scientists could use ML to analyze large-scale, individual-level data to identify or predict who (individuals or subpopulations) is most likely (or least likely) to engage or respond to the intervention [26,27]. Specifically, the application of ML in the preimplementation stage could assist with the selection of the settings or actors, refinement of implementation strategies, and decisions about support activities. ML techniques could predict which sites, practitioners, or target populations will most likely respond well to certain implementation strategies (such as a training session or a health information technology tool), are most likely to need extra support, or might respond better to different strategies. These analyses could be based on prior engagement with strategies that led to increased adoption of EBIs or known characteristics of community (eg, census and environmental health), health systems (eg, geographic location), providers (eg, years of practice), patients (eg, race and ethnicity), and other targeted users.

There are currently no studies using ML approaches to tailor implementation strategies or support needs in the preimplementation stage. A few studies have used unsupervised statistical learning methods, such as latent class analysis and latent profile analysis [28], to identify subgroups of health care providers [27] and patients [26] responding differently to implementation strategies that promote provider-patient communication on critical illness or patients’ physical activities for weight reduction. For example, one study identified 3 groups (or phenotypes) of oncologists based on demographics, practice patterns, and patient panel information [27]. These phenotypes showed different responses to an EHR-based intervention (EHR nudges) aimed at improving advance care planning (ACP) discussion. Oncologists with the lowest volume of patients and a higher rate of baseline ACP discussion showed the greatest improvement compared to those with higher volume or lowest baseline ACP and intermediate volume or baseline ACP. One study used a supervised learning model to identify areas where the implementation of HIV prevention programs should be prioritized. Using state surveillance data on substance use, sexually transmitted diseases, and community characteristics (eg, percent living in poverty), ML modeling identified high-priority areas, of which 79% did not have implemented syringe services programs [29]. Similar modeling approaches could be used to better identify who will adopt what implementation strategies with what supports and tailor resource allocation before an implementation program is launched to improve the adoption and sustainability of EBIs.

Further, ML applications during the setting the stage could also facilitate monitoring when interventions are needed. For instance, using continuously collected clinical or public health data and ML-based phenotyping methods [27], it is possible to prioritize target populations who need the EBIs most at different time points or stages of the implementation of an intervention. Modeling could also trigger notifications to local clinics and public health departments about changes in quality metrics that require improvement, the resources needed to make an improvement (eg, additional staff), or changes in an environmental context (eg, climate change) [30] that could impact disease incidences and health care needs.

### Active Implementation

During the active implementation stage, strategies and support activities are implemented to promote the adoption of an EBI (eg, disease surveillance, prescribing shingles vaccination, and lung cancer screening). During this stage, ML techniques could be incorporated as an implementation strategy. ML-based algorithms relating to the active implementation stage are currently being used to support making accurate diagnoses, disease risk estimation and surveillance, public health campaigns, and clinical decision-making. One example is the use of an ML model to identify foodborne illness in real time (FINDER). This model was developed, implemented, and tested in 2 US cities. FINDER would provide a daily list of restaurants identified as unsafe (likely to have health code violation). Health departments would then conduct an inspection in the restaurants identified by FINDER. The model identified accurately more unsafe restaurants than the previous system or reported complaints [31]. Examples in palliative care include a deep learning model that incorporates patients’ EHR data to predict mortality (those patients most likely to die within 3-12 months). The model-generated estimates were used to inform providers’ care recommendations and decisions about referring patients to palliative care [32,33]. In the context of cancer screening, ML models based on reinforcement learning or ensemble learning are being developed to more accurately identify patients with high risk of cancer [34,35]. These models could be used for cancer screening to balance the benefits of early detection and the costs of overscreening.

Further, in clinical care, clinical decision support (CDS) tools [36,37], including EHR alerts, are common implementation strategies used to promote guideline-concordant practice. ML can be used to develop “smarter” CDS tools to reduce alert fatigue. For example, an ML model was developed to predict whether a provider would respond to shingles vaccination alerts based on the provider’s characteristics (eg, demographics and clinical roles), patient’s demographics, and history of the provider’s interaction with the alerts [38]. The ML model was shown to reduce over 45% of shingles vaccination alerts without reducing weekly shingles vaccination orders [38].

### Monitor, Support, and Sustain

This stage focuses on activities that ensure the sustainability of an intervention. During the monitor, support, and sustain stage, ML can inform changes needed to ensure the adoption and sustainability of practice changes. ML-based methods can leverage vast amounts of data to inform more flexible and adaptive implementation strategies. ML can also facilitate the evaluation and adaptation of strategies and inform where deimplementation is needed. For instance, ML could be used to identify when public health campaigns have reached saturation, need to be refocused, or are missing the target population. For example, during the COVID-19 pandemic, studies use ML models to identify people at greatest risk for COVID-19 death and who should be prioritized for vaccination. Different studies using different populations showed variations in who should be prioritized in informing local public health efforts [39-42]. For example, in clinical practice, implementation scientists leveraged both EHR audit logs and innovative ML-based approaches to monitor the impact of implementing a tobacco control CDS tool in the EHR system [43-45]. According to the Health Information Portability and Accountability Act (HIPAA) [46] and the 2014 release of the Meaningful Use regulations [47], all the EHRs in the United States are required to implement audit logs to unobtrusively track users’ EHR use. In a recent study, a latent-variable statistical ML model was developed to infer EHR-use activities from EHR audit log data [44]. Specifically, the ML model identified topics from EHR log data, where each topic was represented by a probability distribution of microlevel EHR actions such as loading a flow sheet, viewing a problem list, and using a favorite phrase predefined in EHR. Domain experts (3 physicians and 1 EHR specialist) reviewed these topics (eg, the top-ranked microlevel EHR actions belonging to each topic and example EHR sessions representative for each topic) and assigned an EHR-use activity (eg, visit documentation with record review and address CDS alerts) to each topic. This domain expert–informed model was then applied to EHR logs for 3703 encounters (before CDS implementation: n=2633 and after CDS implementation: n=1070) in 4 cancer clinics to monitor changes in providers’ EHR-use between 2019 and 2020 [45]. This study found that clinicians spent more time addressing CDS (more than 32-35 seconds) during a patient visit after CDS implementation (vs before CDS implementation), with compensatory unintended reductions in time spent reviewing patient vital data (less than 61 seconds) and modifying EHR (less than 7-24 seconds) [45]. These findings pointed to potential adaptations of the CDS to improve efficacy and reduce burden [43]. These data-driven findings can inform qualitative studies that aim to understand the causes of the unintended consequences and further inform the decision on refining or deimplementing certain features of the CDS tool.

In summary, despite very few real-world applications of ML in implementation science, there are many options and opportunities to use ML at different stages of implementation; however, some factors are important to take into consideration.

## What Are the Factors to Consider in Using ML for Implementation Science?

As illustrated earlier, ML applications can potentially benefit implementation science across each of the SIF stages. However, many factors can impact the use or validity of these ML-based applications in real-world settings, including achieving equitable outcomes across multiple settings or subpopulations [48].

There are various techniques used in ML [49]. Supervised learning methods can be used to build predictive models (eg, prediction of patients’ risks in illness or poor prognosis and responses of community members, patients, or providers to EBIs and implementation science strategies). Unsupervised learning methods can be used to mine data to identify patterns (eg, identify subgroups of population, patients, and health systems who have different responses to EBIs and implementation strategies). A common practice to develop and validate supervised ML models includes two stages: (1) using a data set to develop and validate (ie, internal validation) the model and (2) using a separate data set (obtained from other similar settings or from a withheld sample) to validate (ie, external validation) the developed model [50,51]. In the first stage, the model can be trained or validated through cross-validation or using a random split of the data set (eg, training or development or validation sets). The model’s parameters and hyperparameters are tuned or set using the training and development sets. In the second stage, the model’s performance is further assessed on the external validation set. Different from supervised learning, there is no ground truth (eg, labels for clusters or subgroups identified by unsupervised learning) to validate results from unsupervised learning in a real-world setting. Consequently, the evaluation process for unsupervised learning is less standard than supervised learning, and the choice of evaluation measures often depends on the unsupervised learning algorithms that are used [52,53]. In general, the quality of clustering results can be measured in 2 aspects when no external references (ie, ground truth) are available: coherence (ie, the similarity of objects falling into the same cluster) and separation (ie, the separation between clusters). Manual chart review is also useful or even necessary for qualitatively validating the clustering results in clinical settings [54]. Both supervised and unsupervised models developed on a specific sample or data set may not be readily applicable to other samples or data sets—the issue with generalizing ML models to different settings [55,56]. This issue has important implications on the use of ML in implementation science and requires paying special attention to model design, development, and validation.

The first factor to consider is that implementation strategies can be implemented at multiple levels (eg, state, county, community, population, health systems, clinicians, and patients), which would determine at which level the ML models would be based. Models developed and validated using data from one level (eg, clinic or community) need further validation and adaptation before being used for predicting outcomes at another level (eg, patient) or an intervention implemented at multiple levels [57]. For example, within the setting the stage phase, a model could be developed using clinician and clinic characteristics (eg, specialty, provider type, and clinic geographic location) to predict which clinicians or clinics will be most likely to adopt a CDS tool. This model, however, is unlikely to be sufficient or valid in predicting the adoption of a multilevel intervention that targets both clinicians and patients (using provider nudges via EHR and patient nudges via SMS text messages). Similarly, public health programs (eg, a tobacco control or vaccination program) often use strategies targeting various levels within a public health jurisdiction (eg, individual, city, county, and state). An ML model predicting the adoption or success of such programs needs to take into account multilevel factors.

Second, the setting (eg, type of clinic and social culture of a specific community), its geographic location, and the time period used in validating the ML model are important factors to consider. These contextual factors are important in implementation science as they impact which strategy or combination of strategies are selected to scale up or modify to ensure the adoption and sustainability of EBI. Models that predict the adoption or sustainability of an implementation strategy developed in primary care clinics are unlikely to have an adequate prediction in specialty clinics in the setting the stage phase. Similarly, an ML-based strategy to improve an EBI in a rural community setting will likely need adaptation to be valid in an urban community setting. Additionally, the time period in which the model was developed needs to be taken into account. For instance, ML-based CDS developed prior to the COVID-19 pandemic may be obsolete or invalid after the pandemic in view of the widespread adoption of telehealth.

Third, when using ML models as an implementation strategy for risk prediction, they should be designed to predict the actual targeted outcome rather than the outcome that is easiest to obtain. For example, consider a risk prediction model being used to direct palliative care interventions. It is easier to train an ML-based tool to predict mortality, as a surrogate for palliative care needs, because mortality is less susceptible to measurement error and is available in palliative care medical records [58,59]. However, training an algorithm on mortality may not identify the individuals with high symptomatic or psychosocial needs who would benefit from palliative care the most. Targeting the risk prediction to the outcome that is most likely to matter for the EBI being implemented is imperative.

Finally, it is critically important to develop and validate models with equity in mind. Many of the algorithms developed in medicine are based on trials with nonrepresentative samples [60]. A recent publication examining various race-biased algorithms used for medical risk predictions demonstrated the potentially harmful consequences of biased algorithms [61]. Within implementation science, as noted earlier, strategies may not work for all. ML models validated in a specific population (eg, pediatric patients) within a specific setting (eg, hospital) could be misused and inequitable if used in a different population (eg, Latino pediatric patients receiving care in a community health center). The learning here is that ML-based implementation strategies need to be tested, validated, and adapted to fit the context of the targeted population to ensure health equity.

## What Are the Challenges?

### Overview

Despite the large amount of clinical data and data from pragmatic implementation trials, there are many challenges associated with data access and data quality. Further, the tools and resources needed to extract and preprocess these data for developing ML may not be easily accessible. For example, extracting and harmonizing patient-level data from the EHRs from multiple health systems to develop a preimplementation ML model could be particularly difficult and time-consuming if these health systems have different EHR vendors. Furthermore, the application of ML in implementation science may result in unintended consequences, and issues related to the sustainability and scalability of the model need to be addressed.

### Data: Quality, Availability, and Type

Public health data and information systems vary with regard to data quality, completeness, collection methods by systems, sampling bias, and underreporting [62-64]. In addition, the collection and generation of public health data are often time-consuming, resulting in delays in data reporting. Similarly, clinical-related data, such as EHR or health insurance claims data, are not designed for research and as such may not be collected and recorded in a systematic standard way. For example, comprehensiveness, completeness, and availability of patient demographic information (eg, race or ethnicity), health insurance data, and clinician data vary greatly by health systems and EHR vendors [65-71]. Additionally, some information that can be critical in the accuracy of ML prediction may reside in unstructured data (eg, a scanned PDF and free text of an encounter note) and, therefore, would require additional preprocessing steps, such as natural language processing [72]. Missing clinical-related data are unlikely to be random [70]. Specifically, EHR data come from a combination of clinician notes, test orders and results, documentation of diagnoses, and patient-reported information. The accuracy and completeness of these data are dependent on the source of the information. For example, the history of a cancer diagnosis can be derived from clinician diagnosis, clinical exchange systems, and patient self-reported history. A study linked EHR data with cancer registry to assess the accuracy of cancer diagnosis in the EHR [66]. Authors found that approximately 45% of cases recorded in the registry did not have a cancer history in their EHR. This information may have been in unstructured data such as in encounter notes. Data used for training an ML model may underrepresent certain patient subgroups [71]. For example, the use of insurance claims data excludes patients without health insurance, and these patients are often socioeconomically disadvantaged individuals. Variation in data documentation and completeness impact not only predictor variables used in the ML models but also the outcome variables. For example, predictive models of emergency department admissions using claims data would miss patients who are uninsured and are more likely to rely on the emergency department for care [73]. Moreover, ML models designed to develop an intervention targeting health system, school system, or community-based organization change may require data on staffing, supplies, or organizational capacity, which could be challenging to obtain.

### Potential for Unintended Consequences

ML models, whether designed for predicting disease risk or for supporting clinical care management and decision-making, are susceptible to bias. Bias can be introduced at multiple points in the development and application process of ML [61,74,75]. As noted earlier, data sources and data representativeness (eg, the population, inclusion or exclusion of diseases, comorbidities, and health risk factors) can greatly influence the ML model and consequently the actions based on the ML model. Further, because ML models can generate data for other ML models, bias can be amplified and can lead to unintended consequences [76]. Char et al [77] proposed a framework for examining ethical considerations of ML models in health care settings, which poses questions about the values and ethics at multiple steps of the model development and implementation. This framework can guide decision-making to minimize bias and can promote accountability and transparency in model development.

### Sustainability and Scalability of the Model

Public health interventions and campaigns are moving targets. For instance, climate change is leading public health departments to adapt or develop new initiatives for disaster preparedness efforts, disease surveillance, and carbon footprint reduction [78-80]. For instance, there is growing evidence of the mental health toll of climate-related events [81], yet strategies to monitor and intervene climate-related mental health burden are scarce [78]. Analogously, health care systems are ever-changing [82] as they must adapt to new clinical care guidelines, changes in reimbursement policies, care delivery modality (ie, telemedicine), quality improvement efforts, and local, state, or federal law amendments. For example, in April 2020, the American Society of Colposcopy and Cervical Pathology released new guidelines to provide recommendations on cervical cancer screening frequency and follow-up tests for abnormal cervical cancer results [83]. These guidelines significantly differ from the previous 2012 version [84]. Any implementation strategies designed to facilitate the adoption of the 2012 guidelines became obsolete and needed to be revised. For another example, EHR-based patient portals are efficient systems for communication between patients and health care providers and platforms for health information exchange. These portals can be a platform for patient-centered implementation strategies to improve the uptake of evidence-based practice. Patient portal tools have been used to improve the uptake of ACP or lung cancer screening [85]. Patient portal adoption before the COVID-19 pandemic, however, remained relatively low and varied widely across patient subgroups (eg, by age and socioeconomic status), diminishing the effectiveness of strategies implemented within the portal [86,87]. The need for social distancing and the uptake of telemedicine during the COVID-19 pandemic led to a rise in patient portal use, which could improve the reach of such strategies [88]. The uptake in patient portal during the pandemic was also associated with a rise in “e-visits,” which were communications between patients and clinicians between in-person visits [89,90]. This led to health care systems to bill for these messages following existing federal rules [90,91], which in turn may limit the use of patient portals and impact their effectiveness as an implementation strategy. This example illustrates how the changes in the health care system can impact a specific implementation strategy. Consequently, the reach, adoption, and sustainability of the EBI it aimed to improve are also impacted. These ever-changing systems pose a significant complication when using ML models [92,93]. How frequently should an ML model be adapted or recalibrated to ensure that it has accurate predictions and is unbiased and ethical? This is a critical factor impacting the use of ML in implementation science and across the 3 stages of implementation and remains to be answered by future studies.

## Conclusions

ML can assist with predicting what will work best, for whom, under what circumstances, and with what level of support, or what and when adaptation and deimplementation are needed. However, there are many remaining challenges with integrating ML into various stages of implementation, which require further research and investigation. Tackling these challenges has the potential to render ML as an innovative and useful tool in implementation science in years to come.

## Abbreviations

ACP: advance care planning
CDS: clinical decision support
EBI: evidence-based intervention
EHR: electronic health record
FINDER: foodborne illness in real time
HIPAA: Health Information Portability and Accountability Act
ML: machine learning
RE-AIM: Reach, Effectiveness, Adoption, Implementation, and Maintenance
SIF: strategic implementation framework
